# Clinical Outcomes of Patients With Drug-Resistant Tuberculous Meningitis Treated With an Intensified Antituberculosis Regimen

**DOI:** 10.1093/cid/cix230

**Published:** 2017-05-04

**Authors:** A Dorothee Heemskerk, Mai Thi Hoang Nguyen, Ha Thi Minh Dang, Chau Van Vinh Nguyen, Lan Huu Nguyen, Thu Dang Anh Do, Thuong Thuy Thuong Nguyen, Marcel Wolbers, Jeremy Day, Thao Thi Phuong Le, Bang Duc Nguyen, Maxine Caws, Guy E Thwaites

**Affiliations:** 1 Oxford University Clinical Research Unit, Ho Chi Minh City, Vietnam;; 2 Nuffield Department of Medicine, University of Oxford, United Kingdom;; 3 Pham Ngoc Thach Hospital for Tuberculosis and Lung Disease, and; 4 Hospital for Tropical Diseases, Ho Chi Minh City, Vietnam; and; 5 Liverpool School of Tropical Medicine, United Kingdom

**Keywords:** tuberculous meningitis, tuberculosis, drug-resistance, isoniazid, levofloxacin

## Abstract

**Background:**

Drug-resistant tuberculous meningitis (TBM) is difficult to diagnose and treat. Mortality is high and optimal treatment is unknown. We compared clinical outcomes of drug-resistant and -susceptible TBM treated with either standard or intensified antituberculosis treatment.

**Methods:**

We analyzed the influence of *Mycobacterium tuberculosis* drug resistance on the outcomes of patients with TBM enrolled into a randomized controlled trial comparing a standard, 9-month antituberculosis regimen (containing rifampicin 10 mg/kg/day) with an intensified regimen with higher-dose rifampicin (15 mg/kg/day) and levofloxacin (20 mg/kg/day) for the first 8 weeks. The primary endpoint of the trial was 9-month survival. In this subgroup analysis, resistance categories were predefined as multidrug resistant (MDR), isoniazid resistant, rifampicin susceptible (INH-R), and susceptible to rifampicin and isoniazid (INH-S + RIF-S). Outcome by resistance categories and response to intensified treatment were compared and estimated by Cox regression.

**Results:**

Of 817 randomized patients, 322 had a known drug resistance profile. INH-R was found in 86 (26.7%) patients, MDR in 15 (4.7%) patients, rifampicin monoresistance in 1 patient (0.3%), and INH-S + RIF-S in 220 (68.3%) patients. Multivariable regression showed that MDR (hazard ratio [HR], 5.91 [95% confidence interval {CI}, 3.00–11.6]), *P* < .001), was an independent predictor of death. INH-R had a significant association with the combined outcome of new neurological events or death (HR, 1.58 [95% CI, 1.11–2.23]). Adjusted Cox regression, corrected for treatment adjustments, showed that intensified treatment was significantly associated with improved survival (HR, 0.34 [95% CI, .15–.76], *P* = .01) in INH-R TBM.

**Conclusions:**

Early intensified treatment improved survival in patients with INH-R TBM. Targeted regimens for drug-resistant TBM should be further explored.

**Clinical Trials Registration:**

ISRCTN61649292.

## Summary

This paper describes outcomes of patients with drug-resistant tuberculous meningitis. Mortality was reduced in patients with isoniazid-resistant infection who received intensified antituberculosis treatment. Early detection and treatment of drug resistance is crucial for improved outcomes of tuberculous meningitis.

Tuberculous meningitis (TBM) is the most fatal form of tuberculosis (TB), killing approximately one-third of patients despite antituberculosis treatment [1–4]. Reports on drug-resistant TBM are limited due to the rarity of the disease and the difficulty of isolating mycobacteria from the cerebrospinal fluid (CSF), but resistance prevalence is broadly consistent with background *Mycobacterium tuberculosis* resistance rates [5–8]. The optimal treatment of drug-resistant TBM is unknown.

Isoniazid and rifampicin are the principal drugs in TBM treatment, given throughout the regimen. Isoniazid penetrates the blood–brain barrier effectively and has the highest early bactericidal activity (EBA) of all first-line drugs, killing approximately 95% of mycobacteria in the first 2 days [9]. Despite low penetration into the CSF, rifampicin is thought to be highly active in the days thereafter, against both dividing and persistent mycobacteria. The complementary role of isoniazid and rifampicin is illustrated by the high mortality of patients with confirmed multidrug-resistant (MDR) TBM, which approaches 100% when treated with standard first-line regimens and as low as 40% when second-line regimens are used [8, 10]. Isoniazid resistance has also been associated with mortality in TBM. In US patients, isoniazid-resistant TBM was associated with death (odds ratio, 2.07 [95% confidence interval {CI}, 1.30–3.29]) regardless of human immunodeficiency virus (HIV) status [11]. In Vietnam, isoniazid-resistant TBM was associated with higher mortality (adjusted hazard ratio [HR], 1.78 [95% CI, 1.18–2.66]) in HIV-infected subjects, but not in HIV-uninfected subjects [8, 12].

In a 1:1 randomized double-blind trial, we investigated whether intensified treatment with higher-dose rifampicin and levofloxacin would benefit patients with TBM [13]. Although the trial did not show an overall benefit of intensified treatment, the results suggested reduced mortality in isoniazid-resistant infection (HR, 0.45 [95% CI, .20–1.02], *P* = .06) [14]. Higher doses of rifampicin are associated with higher EBA [15] and may lead to improved intracranial drug exposure [16, 17]. Fluoroquinolones have antituberculosis activity comparable to that of isoniazid [18]. Levofloxacin penetration in the CSF is good, with levels of 70% of plasma levels [19]. In isoniazid-resistant TBM, the addition of these agents to the first-line regimen may substitute for the loss of isoniazid early bacterial killing. In our trial, intensified treatment was initiated at the onset of treatment regardless of the drug resistance profile of the mycobacteria. This subgroup analysis describes the effect of intensified treatment on outcome in drug-resistant TBM.

## METHODS

### Study Design and Participants

Patients were recruited from 2 tertiary referral hospitals in Ho Chi Minh City, Vietnam. The full protocol and primary results are published elsewhere [13, 14]. In short, adults with a clinical diagnosis of TBM were eligible to enter. Exclusion criteria were a positive CSF Gram stain or India ink stain; known or suspected pregnancy; laboratory contraindications to antituberculosis therapy; MDR TBM diagnosed prior to enrollment; and lack of consent. Written informed consent to participate was obtained from all patients or their relatives. The trial was approved by the Oxford Tropical Research Ethics Committee and the institutional review boards of the Hospital for Tropical Diseases and Pham Ngoc Thach Hospital and the Ministry of Health, Vietnam.

### Treatment

Patients were randomized to receive standard treatment or intensified treatment. Standard treatment consisted of isoniazid (5 mg/kg/day), rifampicin (10 mg/kg/day), pyrazinamide (25 mg/kg/day) and ethambutol (20 mg/kg/day) or streptomycin (20 mg/kg/day) for 3 months, followed by rifampicin and isoniazid at the same doses for 6 months. Those previously treated for tuberculosis received additional streptomycin or ethambutol for the first 3 months. Intensified treatment consisted of the standard regimen with an additional, weight-based dose of rifampicin (5 mg/kg/day) to achieve a total dose of 15 mg/kg/day, and levofloxacin (20 mg/kg/day) for the first 8 weeks of treatment. For patients infected with *M. tuberculosis* resistant to rifampicin and/or isoniazid on drug susceptibility testing (DST), treatment was adjusted at the discretion of the treating clinician, guided by the organism susceptibility. In general, isoniazid resistance was treated with a fluoroquinolone (levofloxacin 750 mg and, in 1 case, moxifloxacin 400 mg until treatment end); occasionally an aminoglycoside (kanamycin 20 mg/kg/day for 3 months) was used early in treatment. However, treatment adjustment was not uniform across centers, as the optimal regimens are not known. Consequently, a subset received additional treatment with at least a fluoroquinolone, regardless of randomized allocation, but treatment was not adjusted in all patients. Patients found to have rifampicin-resistant infection were treated with second-line drugs (a fluoroquinolone and an injectable agent, plus at least 3 other active drugs). All patients received adjunctive dexamethasone for the first 6–8 weeks as previously described [1]. HIV-infected patients received antiretroviral treatment according to Vietnamese guidelines [20].

### Assessment of Outcome

The primary outcome was death during 9-month follow-up. Patients were monitored for drug toxicity, neurological deterioration, and other clinical parameters. A secondary outcome was time to first new neurological event or death. New neurological events were defined as occurrence of any of the following adverse events after enrollment: cerebellar symptoms; coma/consciousness deterioration or a fall in Glasgow Coma Scale score by ≥2 points for ≥2 days from the highest previously recorded; cranial nerve palsy; hemiplegia, paraplegia, tetraplegia, or monoplegia; neurological deterioration requiring ventilation; seizures; or cerebral herniation. Disability was also assessed as a secondary outcome based on the 2 simple questions and the modified Rankin score and classified as good outcome, intermediate outcome, severe disability, or death, as previously described [1, 3, 20].

### Investigations

Patients underwent lumbar puncture at baseline. CSF samples were centrifuged at 4000*g* for 15 minutes. Supernatant was removed to leave a 0.5-mL deposit, which was Ziehl-Neelsen stained, Xpert MTB/RIF (Cepheid) tested, and cultured by Bactec MGIT (Becton Dickinson) [21]. All positive cultures were tested for drug susceptibility to rifampicin, isoniazid, streptomycin, and ethambutol using a Bactec MGIT SIRE kit (Becton Dickinson) following manufacturer’s instructions, which contained the following critical concentrations: streptomycin 1.0 µg/mL, isoniazid 0.1 µg/mL, rifampicin 1.0 µg/mL, and ethambutol 5.0 µg/mL.

### Resistance Categories

Resistance was categorized based on the results of MGIT DST performed against rifampicin, isoniazid, on baseline samples (defined as sample taken any time in between 7 days prior to, or 3 days after enrollment) of CSF, but also sputum, gastric fluid, or blood. The following categories were defined:

- MDR: resistance against both isoniazid and rifampicin.
- Rifampicin resistant (RIF-R): resistance against rifampicin, susceptible to isoniazid.
- Isoniazid resistant (INH-R): resistance against isoniazid, susceptible to rifampicin.
- No isoniazid or rifampicin resistance (INH-S + RIF-S): no resistance to isoniazid or rifampicin.

In all categories, resistance to streptomycin and/or ethambutol may be present.

### Statistical Analysis

Baseline characteristics for each of the resistance categories were summarized as median (interquartile range [IQR]) for continuous data and number (percentage) for categorical data. Clinical variables associated with specified resistance categories were assessed by univariate analysis. The Kruskal-Wallis test was used to compare continuous parameters and Fisher exact test for comparisons between categorical parameters. Kaplan-Meier estimates were used to display survival of patients in the defined resistance categories, by randomized treatment arm. Overall survival and the time to the first new neurological event or death were modeled using Cox regression with resistance categories as the main covariate and additional adjustment for the randomized treatment arm, TBM severity grade, and HIV status. The ordinal disability outcome was modeled using a proportional odds logistical regression model and the same covariates. The effect of intensified treatment on survival in the INH-R group was modeled with a Cox regression model stratified by TBM severity grade and HIV status in accordance with the primary trial publication [14]. To dissect the impact of later treatment alterations with fluoroquinolones in INH-R subjects, “fluoroquinolone use” was assigned as a time-dependent covariate (ie, the start day of fluoroquinolone use was defined as study day 1 (baseline) in subjects randomized to intensified treatment, or the study day corresponding to the start of fluoroquinolone administration if it was initiated in the control arm) in an additional Cox regression model, stratified by TBM severity grade and HIV status. This means that fluoroquinolone usage was modeled to only affect the hazard (ie, the rate) of death from the time-point when fluoroquinolone treatment was actually started onward.

All statistical analyses were performed using the statistical software R version 3.0.2.

## RESULTS

### Baseline Characteristics

**Figure 1.**
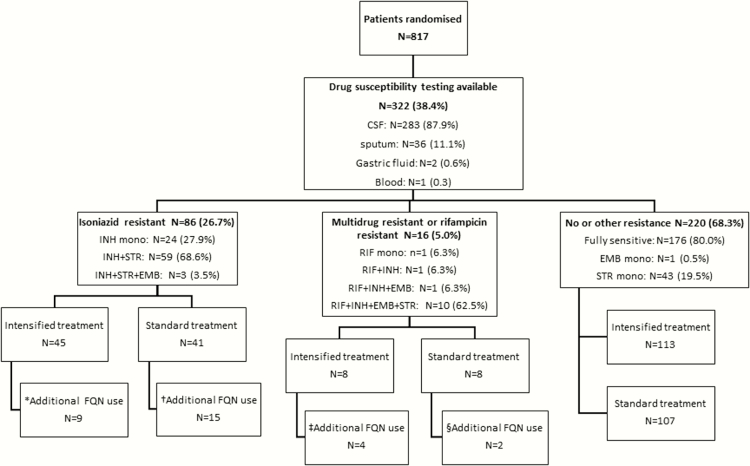
Study flow diagram. *Nine patients who had isoniazid resistance detected and were randomized to intensified treatment received treatment adjustment with the addition of at least a fluoroquinolone. ^†^Fifteen patients who had isoniazid resistance detected and were randomized to standard treatment received treatment adjustment with the addition of at least a fluoroquinolone. ^‡^Four patients who had rifampicin resistance detected and were randomized to intensified treatment received treatment adjustment with at least a fluoroquinolone. For 3 patients, this was part of a second-line treatment schedule and 1 patient received levofloxacin and amikacin added to first-line drugs. ^§^Two patients who had rifampicin resistance detected and were randomized to standard treatment received treatment adjustment with at least a fluoroquinolone. One patient with rifampicin monoresistance received this as part of second-line therapy. The other patient had multidrug resistance and received only additional levofloxacin and no other second-line agents. Abbreviations: CSF, cerebrospinal fluid; EMB, ethambutol; FQN, fluoroquinolone; INH, isoniazid; mono, monoresistance; RIF, rifampicin; STR, streptomycin.

A total of 817 patients were randomized from April 2011 to June 2014. Of those, 322 had DST results for baseline samples: 86 (26.7%) were classified as INH-R, 15 (4.7%) patients as MDR, 1 (0.3%) patient as RIF-R, and 220 (86.3%) patients as sensitive to both drugs. Only 176 (54.7%) patients had fully susceptible infection (Figure 1). Coinfection with HIV was found in 185 (57.5%) patients. Patients in the different resistance categories had a similar clinical presentation, although patients infected with INH-R or MDR were more likely to report a previous episode of TB infection (*P* < .001). In particular, British Medical Research Council (MRC) grade on presentation and chest radiographic findings were similar between groups, and CSF results were also broadly similar. Although not statistically significant, a higher proportion of patients with MDR were HIV infected (13/16 [81.3%]) (Table 1).

**Table 1.** Baseline Characteristics

Characteristic	No or Other Resistance (n = 220)		INH Resistant (n = 86)		RIF Resistant or MDR (n = 16)		All Patients (n = 322)		*P* Value^a^
No. of patients		220/322 (68.3)		86/322 (26.7)		16/322 (5.0)		322/322 (100)	
Age, y, median (IQR)	220	34 (29–41)	86	34 (29–41)	16	34 (31–41)	322	34 (29–41)	.84
Male sex	220	156 (70.9)	86	61 (70.9)	16	14 (87.5)	322	231 (71.7)	.42
Weight, kg, median (IQR)	220	47.7 (43.0–52.0)	86	48.0 (42.1–50.0)	16	50.0 (42.8–52.0)	322	48.0 (43.0–51.0)	.54
Duration of illness, d, median (IQR)	220	15.0 (10.0–30.0)	86	15.0 (10.0–29.5)	16	25.0 (10.3–30.0)	322	15.0 (10.0–30.0)	.68
Previous episode of TB	220	33 (15.0)	86	25 (29.1)	16	8 (50.0)	322	66 (20.5)	<.001
HIV infected	220	125 (56.8)	86	47 (54.7)	16	13 (81.3)	322	185 (57.5)	.13
Cranial nerve palsy	220	52 (23.6)	86	23 (26.7)	16	2 (12.5)	322	77 (23.9)	.51
Hemiplegia	220	31 (14.1)	86	19 (22.1)	16	3 (18.8)	322	53 (16.5)	.23
Paraplegia	220	7 (3.2)	86	9 (10.5)	16	0 (0)	322	16 (5.0)	.03
Quadriplegia	220	4 (1.8)	86	1 (1.2)	16	0 (0)	322	5 (1.6)	1.00
GCS score, median (IQR)	220	14 (11–15)	86	15 (11–15)	16	15 (13–15)	322	14 (11–15)	.40
MRC grade^b^	220		86		16		322		.41
1		87 (39.5)		29 (33.7)		8 (50.0)			
2		83 (37.7)		39 (45.3)		7 (43.8)			
3		50 (22.7)		18 (20.9)		1 (6.2)			
Serum sodium level, mmol/L, median (IQR)	205	126.0 (122.0–131.0)	81	125.0 (121.0–130.0)	16	125.5 (121.0–128.0)	302	126.0 (122.0–131)	.16
Chest radiograph	220		85		16		321		.75
Consistent with TB		124 (56.4)		45 (52.9)		9 (56.3)		178 (55.5)	
Miliary TB		51 (23.2)		16 (18.8)		3 (18.8)		70 (21.8)	
Abnormal other		21 (9.6)		12 (14.1)		1 (6.3)		34 (10.6)	
Normal		24 (10.9)		12 (14.1)		3 (18.8)		39 (12.2)	
CSF results									
WBC count, cells/µL, median (IQR)	216	199.5 (72.8–386)	86	108.5 (32.3–316)	16	253.5 (64.3–340.0)	318	180.0 (58.0–382)	.06
Lymphocytes, %, median (IQR)	210	74.5 (40.0–90.0)	84	86.0 (53.5–100.0)	15	80.0 (34.0–95.0)	309	78.0 (44.0–95.0)	.04
Protein level, g/L, median (IQR)	211	1.48 (1.02–2.38)	82	1.24 (0.81–1.87)	16	1.28 (0.74–2.38)	309	1.41 (0.90–2.30)	.15
Lactate, mmol/L, median (IQR)	199	5.93 (4.50–7.69)	78	5.20 (4.40–6.84)	15	5.50 (4.81–7.30)	292	5.80 (4.49–7.40)	.15
Glucose, mmol/L, median (IQR)	212	1.49 (0.98–2.00)	81	1.58 (1.04–2.11)	16	1.60 (0.95–2.28)	309	1.51 (1.00–2.10)	.52
CSF/blood glucose ratio, median (IQR)	177	0.25 (0.16–0.33)	72	0.25 (0.19–0.34)	15	0.26 (0.18–0.36)	264	0.25 (0.16–0.33)	.69
Ziehl-Neelsen smear positive	210	135 (64.3)	83	45 (54.2)	15	7 (46.7)	308	187 (60.7)	.14
Xpert result positive	202	166 (82.2)	79	62 (78.5)	14	7 (50.0)	295	235 (79.7)	.02
Duration of initial admission, d, median (IQR)	219	30 (23–38)w	86	31 (26–37)	16	30 (12–32)	321	30 (24–37)	.46

Abbreviations: CSF, cerebrospinal fluid; GCS, Glasgow Coma Scale; HIV, human immunodeficiency virus; INH, isoniazid; IQR, interquartile range; MDR, multidrug resistant; MRC, British Medical Research Council; RIF, rifampicin; TB, tuberculosis; WBC, white blood cell.

^a^Summary statistics are frequency (%) for categorical and median (IQR) for continuous variables. *P* values are based on Fisher exact test (categorical data) or the Kruskal-Wallis test (continuous data).

^b^MRC denotes modified British Medical Research Council criteria. Grade 1 indicates a GCS score of 15 with no neurologic signs (baseline); grade 2, a score of 11–14 (or 15 with focal neurologic signs); and grade 3, a score of ≤10.

### Time to Diagnosis of Drug Resistance and Relevant Treatment Adjustment With Fluoroquinolones

The median time to diagnosis of INH-R in the 86 INH-resistant patients was 70 days (IQR, 48–83 days). When drug resistance was detected prior to 56 days, the study intervention was stopped (without unblinding of the allocation), if treatment adjustments were made. Among the 41 subjects in the standard-of-care arm, 15 subsequently had their treatment adjusted, by adding (at least) a fluoroquinolone (levofloxacin, n = 14; moxifloxacin, n = 1) after a median time to initiation of 76 days (IQR, 61–92 days). In the intensified treatment arm (n = 45), 9 INH-R patients received additional fluoroquinolone treatment (levofloxacin), with a median time to treatment adjustment of 105 days (IQR, 66–113 days) (Figure 1). Characteristics for patients receiving adjustment of treatment and those who did not are given in Supplementary Table 1.

Characteristics, outcome, and drug management of patients with MDR TBM are given in Supplementary Table 2. One patient had RIF-R (detected by Xpert MTB/RIF) and received treatment with levofloxacin as part of second-line treatment, 8 days after TBM treatment initiation. Three patients with MDR infection (detected by Xpert MTB/RIF) were treated with additional levofloxacin as part of second-line treatment with a median time to treatment of 10 days (IQR, 6–23 days). Two patients with MDR TBM received additional levofloxacin, but not second-line treatment, 1 in each arm (Figure 1).

### Outcome

**Figure 2.**
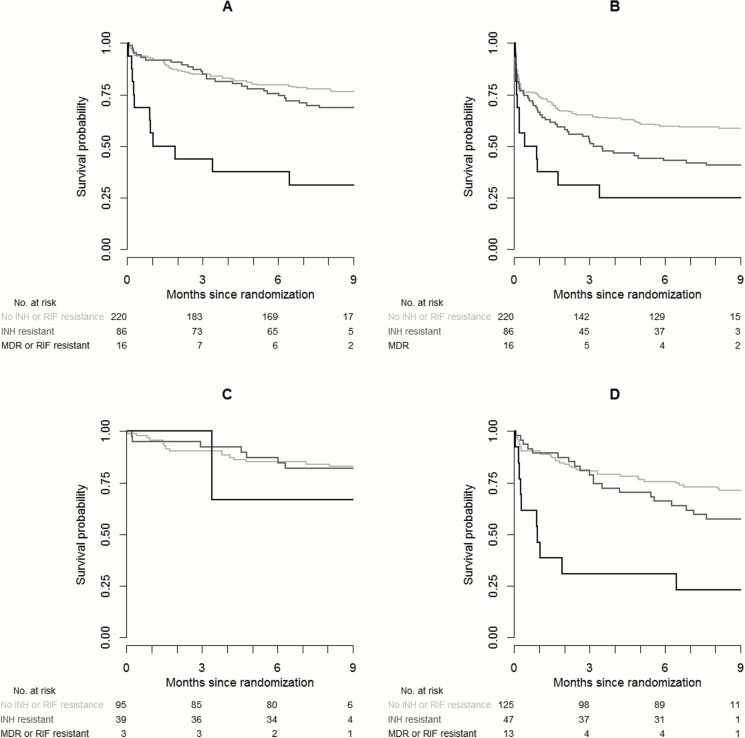
Time-to-event analysis by resistance category and human immunodeficiency virus (HIV) status. *A*, Kaplan-Meier (KM) estimates of survival during 9 months by resistance category. *B*, KM estimates of combined endpoint of time to new neurological event or death by resistance category. *C*, KM estimates of survival by resistance category of HIV-uninfected patients. *D*, KM estimates of survival by resistance category of HIV-infected patients. The light gray line represents patients with tuberculous meningitis (TBM) with no isoniazid (INH) or rifampicin (RIF) resistance. The darker gray line represents patients with TBM with INH resistance, but no RIF resistance. The black line represents patients with TBM with RIF resistance or multidrug resistance. Abbreviations: INH, isoniazid; MDR, multidrug resistance; RIF, rifampicin.

Overall, 90 of 322 (28.0%) patients died during follow-up: 27 of 86 (31.4%) in the INH-R category, 11 of 16 (68.8%) in the MDR/RIF-R group, and 52 of 220 (23.6%) in the INH-S + RIF-S category (Figure 2A). Multivariable Cox regression identified HIV infection (HR, 2.60 [95% CI, 1.62–4.17], *P* < .001), disease severity grade (HR, 1.07 [95% CI, .62–1.84] for grade 2 vs 1; HR, 4.53 [95% CI, 2.71–7.59] for grade 3 vs 1; overall *P* < .001) and MDR infection (HR, 5.91 [95% CI, 3.00–11.64], *P* < .001) as independent predictors of death, but not intensified treatment (HR, 0.92 [95% CI, .60–1.40], *P* = .70) or INH-R (HR, 1.30 [95% CI, .81–2.07], *P* = .28), consistent with previous predictors [14].

Of 322 patients, 154 (47.8%) patients met the combined endpoint of new neurological event and death: 64 (19.9%) neurological events in survivors, 69 (21.4%) neurological events with subsequent death, and 21 (6.5%) deaths in patients without a prior recorded neurological event. Adjusted Cox regression showed a significant effect of isoniazid resistance on the occurrence of any new neurological event or death combined (HR, 1.58 [95% CI, 1.11–2.23], *P* = .01) (Figure 2B).

Mortality in HIV-infected patients was increased (Figure 2C and D). Although mortality appeared higher in INH-R compared to those without resistance in this subgroup, the comparison did not reach statistical significance (adjusted HR, 1.48 [95% CI, .86–2.57], *P* = .16) (Figure 2D).

There was a nonsignificant trend toward worse disability at 9 months in the INH-R category (cumulative OR, 1.44 [95% CI, .90–2.32], *P* = .13). Three of 5 survivors in the MDR/RIF-R group were left severely disabled (Table 2).

**Table 2.** Disability Status at 9 Months by Resistance Category

Disability	No or Other Resistance (n = 220)	INH Resistant (n = 86)	RIF Resistant or MDR (n = 16)	Cumulative Odds Ratio^a^ (95% CI)	*P* Value
Good	91 (41.4)	31 (36.0)	2 (12.5)	INH: 1.44	
Intermediate	53 (24.1)	17 (19.8)	0 (0)	(.90–2.32)	.13
Severe	20 (9.1)	11 (12.8)	3 (18.8)	RIF MDR: 10.31	
Death	52 (23.6)	27 (31.4)	11 (68.8)	(3.39–31.36)	<.001

Data are presented as No. (%) unless otherwise indicated.

Abbreviations: CI, confidence interval; INH, isoniazid; MDR, multidrug resistance; RIF, rifampicin monoresistance.

^a^Cumulative odds ratios for INH and RIF MDR (compared to no or other resistance) were obtained from a proportional odds logistical regression model with the resistance category as the main covariate and adjustment for human immunodeficiency virus status, tuberculous meningitis severity grade, and randomized treatment arm.

Seventy-eight INH-R isolates were available for molecular analysis: 70.5% had mutations in the KatG region (associated with high-level resistance), 19.2% had mutations in the inhA promoter region (associated with low-level resistance), and 12.8% had no mutation identified. Survival by resistance mutation is shown in Supplementary Figure 1. Resistance mutations were not associated with mortality by Cox regression (adjusted HR, 0.80 [95% CI, .31–2.06], *P* = .66).

### Intensified Treatment

**Figure 3.**
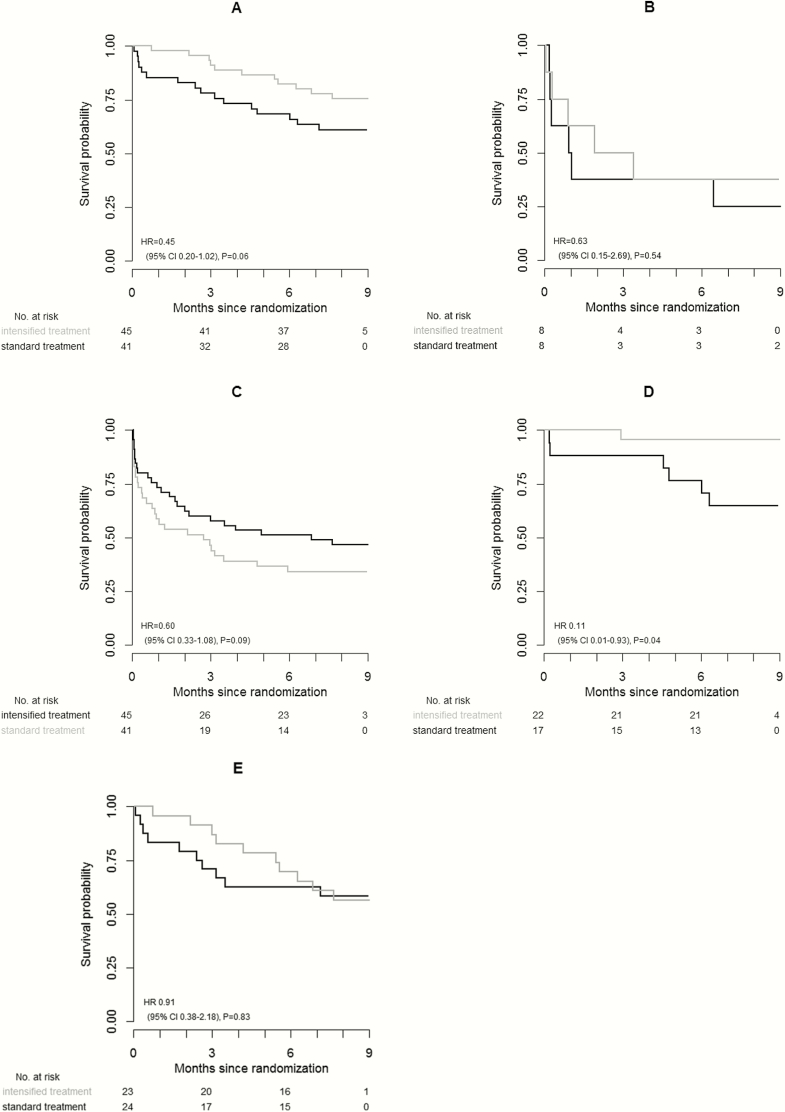
Time-to-event analysis by randomized treatment. *A*, Overall 9-month survival by randomized arm of 86 patients in the isoniazid-resistant, rifampicin-susceptible (INH-R) category. *B*, Overall 9-month survival by randomized treatment arm of 16 patients in the rifampicin multidrug resistance category. *C*, Time to new neurological event or death by randomized treatment of 86 patients in the INH-R category. *D* and *E*, Overall 9-month survival by randomized treatment of 39 human immunodeficiency virus (HIV)–uninfected (*D*) and 47 HIV-infected (*E*) patients in the INH-R category. Cox regression was stratified by tuberculous meningitis severity grade and HIV status. Abbreviations: CI, confidence interval; HR, hazard ratio.

The overall effect of randomized intensified treatment on survival in INH-R and MDR/RIF-R is shown in Figure 3. In INH-R TBM, when stratified by disease severity and HIV status, the HR of death of intensified treatment vs standard treatment was 0.45 (95% CI, .20–1.02, *P* = .06). For the combined outcome of new neurological event or death in INH-R patients, the stratified HR of intensified treatment was 0.60 (95% CI, .34–1.08, *P* = .09) (Figure 3C). Intensified treatment appeared to be predominantly beneficial in HIV-uninfected patients with isoniazid-resistant infection (Figure 3D and 3E). Of the HIV-uninfected patients, 6 of 17 (35.3%) died in the standard treatment arm, compared with 1 of 22 (4.6%) in the intensified treatment arm (HR, 0.11 [95% CI, .01–.93], *P* = .04) (Figure 3D). In contrast, 10 of 24 (41.7%) of HIV-infected patients with isoniazid resistance died in the standard treatment arm, compared with 10 of 23 (43.5%) in the intensified treatment arm (HR, 0.91 [95% CI, .38–2.18], *P* = .83) (Figure 3E).

### Effect of Treatment Adjustment for INH-R

To extricate the effect of treatment adjustments on the over- or underestimation of the effect of early intensified treatment in INH-R TBM, we performed explorative analyses accounting for these late adjustments. Two different Cox regression models were created, including “fluoroquinolone use” as a time-dependent covariate, as initiation of treatment adjustment is dependent on survival up to drug resistance detection. Within the INH-R group, 60 patients received treatment with at least a fluoroquinolone either as part of intensified treatment (n = 45, early) or as targeted INH-R treatment (n = 15, late), while 26 patients did not receive any intensified or adjusted treatment throughout 9 months of treatment. Of those receiving intensified treatment or adjustment with a fluoroquinolone, 15 of 60 (25.0%) died. In the standard treatment group, with no treatment adjustment, 12 of 26 (46.1%) patients died. Cox regression showed a significant benefit of overall “fluoroquinolone use” on survival (HR, 0.38 [95% CI, .18–.80], *P* = .01), stratified by disease severity grade and HIV infection. A significant benefit of later adjustment only could not be established in this population (HR, 0.59 [95% CI, .18–1.91], *P* = .38) (Table 3). Furthermore, the second model showed a more pronounced effect on the rate of death for immediate treatment (HR, 0.34, affecting survival from the time of randomization onward) than of subsequent initiation (HR, 0.59, affecting survival only from the time of fluoroquinolone treatment initiation onward), suggesting that earlier fluoroquinolone treatment is better.

**Table 3.** Effect of Intensified and Adjusted Treatment in Isoniazid-Resistant, Rifampicin-Susceptible Tuberculous Meningitis

Treatment	Hazard Ratio (95% CI)	*P* Value
Fluoroquinolone use at any time point^a^		
Intensified or adjusted treatment (Tdc)	0.38 (.18–.80)	.01
MRC grade		
1	1 (reference category)	
2	1.60 (.67–3.82)	.29
3	2.96 (1.11–7.89)	.03
HIV infection		
Uninfected	1 (reference category)	
Infected	2.93 (1.31–6.59)	.01
Effect of later adjustments^b^		
Late adjustment regimen (Tdc)	0.59 (.18–1.91)	.38
Randomized arm		
Standard treatment	1 (reference category)	
Intensified treatment	0.34 (.15–.76)	.01
MRC grade		
1	1 (reference category)	
2	1.57 (.66–3.75)	.31
3	3.03 (1.14–8.08)	.03
HIV infection		
Uninfected	1 (reference category)	
Infected	2.82 (1.25–6.39)	.01

Abbreviations: CI, confidence interval; HIV, human immunodeficiency virus; MRC, Medical Research Council; Tdc, time-dependent covariate.

^a^Cox regression including treatment enrichment with a fluoroquinolone as a time-dependent covariate, adjusted for tuberculous meningitis (TBM) severity grade (MRC grade) and HIV status.

^b^Cox regression including only late treatment adjustment as a time-dependent covariate, while treatment allocation is treated as a baseline covariate, corrected for TBM severity grade and HIV status. Eighty-six patients in the isoniazid-resistant, rifampicin-susceptible category were included in the analysis.

### Multidrug-Resistant TBM

In total, 15 patients had MDR and 1 patient had RIF-R TBM. The median time to death of MDR patients was 27 days (IQR, 7–45 days). In the MDR/RIF-R group combined, 4 patients received second-line treatment according to the Vietnamese TB program guidelines (including an injectable aminoglycoside, kanamycin 20 mg/kg/day; a fluoroquinolone, levofloxacin 750 mg/day; pyrazinamide 25 mg/kg/day; and at least 2 of ethionamide 15–20 g/kg/day, prothionamide 15–20 mg/kg/day, cycloserine 15–20 mg/kg/day, or para-aminosalicylic acid 8 g/day). Four patients receiving second-line treatment and 1 patient on standard treatment survived 9 months. All other patients died, with a median time to death of 27 days (IQR, 7–45 days). A summary of individual patient characteristics and treatment are given in Supplementary Table 2. The sample size was too small to detect potential efficacy of intensified treatment (HR, 0.63 [95% CI, .15–2.69], *P* = .54) (Figure 3B).

## DISCUSSION

To date, no clinical trials have explored optimal treatment regimens for drug-resistant TBM. In our blinded randomized controlled trial of intensified antituberculosis treatment for TBM, intensified treatment led to reduction of mortality in INH-R infection. This finding is crucial, as isoniazid resistance is increasingly prevalent and associated with worse outcome. Improved treatment will impact mortality and morbidity of TBM. A considerable proportion of baseline isolates were INH-R (26.7%), reflecting the high incidence of isoniazid-resistant TB in Vietnam.

We performed a time-dependent Cox regression, to correct for relevant treatment adjustments made for drug-resistant infection. This showed that treatment benefit in INH-R was mainly generated by the early randomized intervention. We could not establish whether fluoroquinolones improved outcomes from INH-R TBM when added later in treatment based on DST, but our findings underscore the necessity for improved early drug resistance detection. The use of rapid molecular testing of pulmonary TB by Xpert MTB/RIF has facilitated early diagnosis of rifampicin resistance and second-line treatment initiation [22]. Xpert MTB/RIF has been recommended by the World Health Organization for direct use on CSF as the initial diagnostic test [21, 23]; however, isoniazid resistance cannot be detected by the current Xpert cartridge. The line-probe assays, such as Genotype MTBDR (Hain Life-Science, Nehren, Germany), can detect both rifampicin and isoniazid resistance. Although the bacillary load in CSF is likely too low for direct use, these assays may be used on bacteria cultured from CSF samples, which should reduce time to resistance detection.

Eleven of 16 (68.8%) of those with MDR/RIF-R TBM died by 9 months. Of the 5 survivors, 4 received early second-line treatment, based on the results of Xpert MTB/RIF resistance testing. Early diagnosis of MDR TBM allows the instigation of second-line regimens, but few data are available to guide regimen tailoring.

The limitations of this study include the potential for missing patients with drug-resistant disease, due to the poor sensitivity of CSF culture. Only 39.4% (322/817) had a known drug resistance profile; the impact of resistance on the remaining patients could not be assessed. Positive CSF culture may be linked to higher bacterial loads and worse outcomes, which may confound our findings. We had no minimum inhibitory concentrations or pharmacokinetic data for isoniazid. Higher-dose rifampicin and fluoroquinolone were given jointly as 1 intervention; hence, it is impossible to tease the effect of both drugs apart. Higher doses of rifampicin have been associated with improved outcome in an Indonesian study [24]. In addition, the impact of later (nonrandomized) fluoroquinolone treatment on outcome is hard to assess given the small numbers and the potential for differences in disease severity and postrandomization management to confound the analysis.

In conclusion, early intensified treatment increased survival in patients with INH-R TBM. Early detection of drug-resistant TBM is key to improving outcomes.

## Supplementary Data

Supplementary materials are available at *Clinical Infectious Diseases* online. Consisting of data provided by the authors to benefit the reader, the posted materials are not copyedited and are the sole responsibility of the authors, so questions or comments should be addressed to the corresponding author.

## Supplementary Material

DRTBM_Supplementary_file_13FEB17

## Notes

***Disclaimer.*** The funders played no role in the design, interpretation, or publication of the study.

***Financial support.*** This work was supported by the Wellcome Trust, United Kingdom (091925 Z/10/Z and 081814/Z/06/Z), and the Li Ka Shing Foundation, Hong Kong (LG36).

***Potential conflicts of interest.*** All authors: No reported conflicts of interest. All authors have submitted the ICMJE Form for Disclosure of Potential Conflicts of Interest. Conflicts that the editors consider relevant to the content of the manuscript have been disclosed.
