# Supplementary material for: Clinical Outcomes of Patients With Drug-Resistant Tuberculous Meningitis Treated With an Intensified Antituberculosis Regimen
Source: Clin Infect Dis. 2017 May 4;65(1):20–8. doi: 10.1093/cid/cix230 (PMC5850451; doi:10.1093/cid/cix230)
Supplement: DRTBM_Supplementary_file_13FEB17 [file cix230_suppl_drtbm_supplementary_file_13feb17.docx]

**Supplementary file**


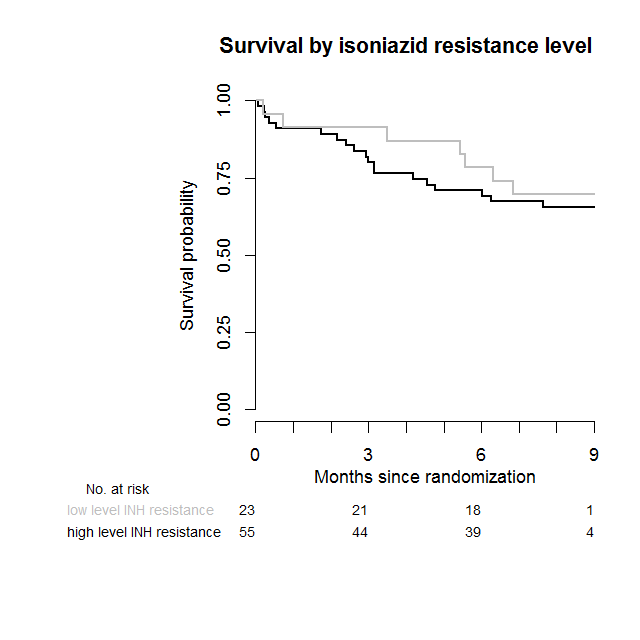


HR 0.80

(95%CI 0.31-2.06, P=0.66)

Figure S1. Survival by isoniazid resistance mutation

From patients with isoniazid resistance, 78 isolates were available for molecular analysis of resistance mutations. 55/78 Isolates (70.5%) had mutation in the KatG region, 15/78 (19.2%) had mutation in the inhA promoter region. 10/78 (12.8%) had no mutation identified. There were dual mutations in both inhA and KatG regions in three isolates. In order to estimate whether there was a survival benefit associated with resistance mutations and estimated resistance level, dual mutations were classified as KatG (associated with high level resistance) and unknown mutations were grouped with inhA (associated with intermediate level resistance).

“Low level resistance” (grey line) is defined as inhA mutations and unknown resistance mutations.

“High level resistance” (black line) is defined as mutations in KatG region.

Hazard ratio (HR) adjusted for TBM severity grade and HIV infection

|  |  | All patients (N=86) |  | No treatment adjustment (N=62) |  | Treatment adjustment (N=24) | Comparison* |
| --- | --- | --- | --- | --- | --- | --- | --- |
| Characteristic | n | Summary statistic | n | Summary statistic | n | Summary statistic | (P-value) |
| Time to death (days) | 86 | 270 (185,271) | 62 | 270 (132,271) | 24 | 270 (268,271) | 0.65 |
| 9 month survival | 86 |  | 62 |  | 24 |  | 0.08 |
| - survived |  | 59(68.6%) |  | 39(62.9%) |  | 20(83.3%) |  |
| - died |  | 27(31.4%) |  | 23(37.1%) |  | 4(16.7%) |  |
| 9 month disability | 86 |  | 62 |  | 24 |  | 0.30 |
| - good |  | 31(36.1%) |  | 21(33.9%) |  | 10(41.7%) |  |
| - intermediate |  | 17(19.8%) |  | 11(17.7%) |  | 6(25.0%) |  |
| - severe |  | 11(12.8%) |  | 7(11.3%) |  | 4(16.7%) |  |
| - death |  | 27(31.4%) |  | 23(37.1%) |  | 4(16.7%) |  |
| Randomised arm | 86 |  | 62 |  | 24 |  | 0.10 |
| - Standard |  | 41(47.7%) |  | 26(41.9%) |  | 15(62.5%) |  |
| - Intensified |  | 45(52.3%) |  | 36(58.1%) |  | 9(37.5%) |  |
| Sex | 86 |  | 62 |  | 24 |  | 0.79 |
| - female |  | 25(29.1%) |  | 19(30.7%) |  | 6(25.0%) |  |
| - male |  | 61(70.93%) |  | 43(69.35%) |  | 18(75.0%) |  |
| Age (years) | 86 | 34 (29,41) | 62 | 33(27,40) | 24 | 34(31,41) | 0.43 |
| Weight (kg) | 86 | 48.0(42.1,50.0) | 62 | 47.0(42.1,50.0) | 24 | 50.00(42.3,54.3) | 0.30 |
| Previous TB episode | 86 |  | 62 |  | 24 |  | 0.79 |
| - no |  | 61(70.9%) |  | 43(69.4%) |  | 18(75.0%) |  |
| - yes |  | 25(29.1%) |  | 19(30.7%) |  | 6(25.0%) |  |
| Duration of illness (days) | 86 | 15 (10,30) | 62 | 15(10,30) | 24 | 15(10,21) | 0.67 |
| Hemiplegia | 86 |  | 62 |  | 24 |  | 1.00 |
| - no |  | 67(77.9%) |  | 48(77.4%) |  | 19(79.2%) |  |
| - yes |  | 19(22.1%) |  | 14(22.6%) |  | 5(20.8%) |  |
| Paraplegia | 86 |  | 62 |  | 24 |  | 1.00 |
| - no |  | 77(89.5%) |  | 55(88.7%) |  | 22(91.7%) |  |
| - yes |  | 9(10.5%) |  | 7(11.3%) |  | 2(8.3%) |  |
| Quadriplegia | 86 |  | 62 |  | 24 |  | 1.00 |
| - no |  | 85(98.8%) |  | 61(98.4%) |  | 24(100%) |  |
| - yes |  | 1(1.2%) |  | 1(1.6%) |  | 0(0%) |  |
| Baseline GCS | 86 | 15 (11,15) | 62 | 15(11,15) | 24 | 14(13,15) | 0.93 |
| MRC grade† | 86 |  | 62 |  | 24 |  | 0.86 |
| - 1 |  | 29(33.7%) |  | 21(33.9%) |  | 8(33.3%) |  |
| - 2 |  | 39(45.4%) |  | 27(43.6%) |  | 12(50.0%) |  |
| - 3 |  | 18(20.9%) |  | 14(22.6%) |  | 4(16.7%) |  |
| Chest Xray result | 85 |  | 61 |  | 24 |  | 0.69 |
| - consistent TB |  | 45(52.9%) |  | 32(52.5%) |  | 13(54.2%) |  |
| - miliary TB |  | 16(18.8%) |  | 10(16.4%) |  | 6(25.0%) |  |
| - other lesions |  | 12(14.1%) |  | 10(16.4%) |  | 2(8.3%) |  |
| - normal |  | 12(14.1%) |  | 9(14.8%) |  | 3(12.5%) |  |
| Admission hospital | 86 |  | 62 |  | 24 |  | 0.05 |
| - HTD |  | 38(44.2%) |  | 23(37.1%) |  | 15(62.5%) |  |
| - PNT |  | 48(55.8%) |  | 39(62.9%) |  | 9(37.5%) |  |
| HIV status | 86 |  | 62 |  | 24 |  | 0.81 |
| - negative |  | 39(45.4%) |  | 29(46.8%) |  | 10(41.7%) |  |
| - positive |  | 47(54.7%) |  | 33(53.2%) |  | 14(58.3%) |  |

Table S1. Characteristics of INH-R patients who received treatment adjustment or no adjustment

*Summary statistics are frequency (percentage) for categorical and median (IQR) for continuous variables. P-values are based on Fisher’s exact test (categorical data) or the Kruskal-Wallis test (continuous data).

†MRC denotes modified British Medical Research Council criteria. Grade 1 indicates a Glasgow coma (GCS)score of 15 with no neurologic signs, grade 2 a score of 11 to 14 (or 15 with focal neurologic signs), and grade 3 a score of 10 or less. Of note, the MRC grade at study enrolment is reported here.

INH=isoniazid, HTD=Hospital for Tropical Diseases, PNT=Pham Ngoc Thach Hospital for Tuberculosis and Lung Diseases

| ID | Time to death (days) | Disability outcome | Randomised treatment | Sex | Age | Previous TB | MRC grade | CD4 cell count (/mm^3^) | HIV status | ARV status | SM | INH | RIF | Xpert RIF detection | EMB | Category | Treatment regimen |
| --- | --- | --- | --- | --- | --- | --- | --- | --- | --- | --- | --- | --- | --- | --- | --- | --- | --- |
| 1 | 58 | death | Intensified | male | 28 | yes | 1 | 8 | positive | not on ARV | Res | Res | Res | *NA* | Res | MDR | 23dRHZE*'/35dSZH |
| 2 | 8 | death | Standard | male | 33 | yes | 2 | 5 | positive | not on ARV | Res | Res | Res | *NA* | Res | MDR | 8dRHZSE |
| 3 | 5 | death | Standard | male | 35 | yes | 1 | 34 | positive | not on ARV | Res | Res | Res | Res | Res | MDR | 6dHRZSE |
| 4 | 269 | good | Intensified | male | 31 | no | 1 | 57 | positive | on ARV | Res | Res | Res | Res | Res | MDR | 8dHRZE/2EKmLfxZEthCSH/ 4KmLfxEthCs/2LfxEthCs |
| 5 | 1 | death | Intensified | male | 39 | yes | 2 | 305 | positive | on ARV | sens | Res | Res | Res | sens | MDR | 1dRHZSE |
| 6 | 289 | severe | Standard | male | 34 | yes | 2 | 15 | positive | on ARV | sens | sens | Res | Res | sens | RIF-r | 8dHRZSE/>9EZKmLfxCSPto |
| 7 | 271 | severe | Intensified | male | 45 | yes | 2 | 214 | positive | on ARV | Res | Res | Res | *NA* | Res | MDR | 3HRZSE/>6KmLfxEZPtoCs |
| 8 | 103 | death | Intensified | male | 47 | yes | 1 | *NA* | negative | *NA* | Res | Res | Res | *NA* | Res | MDR | 39dRHZSE*/1RHZSE |
| 9 | 289 | severe | Standard | male | 53 | yes | 1 | *NA* | negative | *NA* | sens | Res | Res | *NA* | Res | MDR | 37dRHZSE |
| 10 | 31 | death | Standard | male | 35 | no | 1 | 23 | positive | on ARV | Res | Res | Res | Res | sens | MDR | 28dRHZE/3dLfx |
| 11 | 28 | death | Standard | female | 29 | no | 1 | 55 | positive | on ARV | Res | Res | Res | ind | Res | MDR | 28dRHZE |
| 12 | 27 | death | Intensified | male | 31 | no | 1 | 11 | positive | not on ARV | Res | Res | Res | *NA* | Res | MDR | 27dRHZE |
| 13 | 6 | death | Standard | male | 32 | no | 2 | 25 | positive | not on ARV | Res | Res | Res | *NA* | Res | MDR | 6dRHZE |
| 14 | 196 | death | Standard | female | 33 | no | 2 | 10 | positive | not on ARV | Res | Res | Res | *NA* | Res | MDR | 46dRHZE |
| 15 | 9 | death | Intensified | male | 25 | no | 3 | 48 | positive | not on ARV | Res | Res | Res | Res | sens | MDR | 6dRHZEAmkLfx |
|  | 267 | good | Intensified | male | 49 | no | 2 | *NA* | negative | *NA* | Res | Res | Res | *NA* | sens | MDR | 1RHZSE/4KmLfxZECsEthPas/2KmLfxECsEthPas/  >2KmLfxECsPas |

Table S2. Summary of patients with multidrug resistant or rifampicin mono-resistant TBM

ARV=antiretroviral treatment, S or SM=streptomycin, H or INH=isoniazid, R or RIF=rifampicin, E or EMB=ethambutol, MDR=multidrug resistant, Z=pyrazinamide, Km=kanamycin, Lfx=levofloxacin, Eth=ethionamide, Cs=cycloserine, Pto=prothionamide, Pas=para-aminosalicylic acid, Res=resistant, sens=sensitive, ind=indeterminate, NA=data missing
